# Association between a Deficit Accumulation Frailty Index and Mobility Outcomes in Older Adults: Secondary Analysis of the Lifestyle Interventions and Independence for Elders (LIFE) Study

**DOI:** 10.3390/jcm9113757

**Published:** 2020-11-22

**Authors:** Joshua D. Brown, Golnoosh Alipour-Haris, Marco Pahor, Todd M. Manini

**Affiliations:** 1 Center for Drug Evaluation & Safety, Department of Pharmaceutical Outcomes & Policy, University of Florida College of Pharmacy, Gainesville, FL 32610, USA; g.alipourharis@ufl.edu; 2 Institute on Aging, University of Florida College of Pharmacy, Gainesville, FL 32610, USA; mpahor@ufl.edu (M.P.); tmanini@ufl.edu (T.M.M.)

**Keywords:** mobility, disability, frailty, deficit accumulation, LIFE Study, older adults, healthy aging

## Abstract

Frailty is a geriatric syndrome represented by susceptibility to precipitating health events and reduced functional reserve. Frailty can be difficult to measure in clinical practice and research. One approach to approximate frailty is based on a deficit accumulation approach, which assesses a larger number of less specific measures such as the presence of comorbidities, physical or cognitive assessments, and lab tests, and summarizes these as a frailty index. The objective of this study was to develop such an index using the Lifestyle Interventions and Independence for Elders (LIFE) Study and evaluate the validity of the frailty measure derived based on baseline information via its association with the primary outcomes of the trial, namely major mobility disability (MMD) and persistent MMD (pMMD). Further, this study aimed to evaluate the effectiveness of the physical activity intervention among participants based on their baseline frailty score. Subjects in the LIFE Study were evaluated at baseline for demographics, clinical history, and a battery of physical and cognitive functioning assessments. In total, 75 possible deficits were scored either as present (yes/no) or based on each score’s quintiles for score-based assessments. The frailty index was measured as the total sum of deficits divided by the total number of possible deficits on a continuous scale between 0 and 100 (i.e., percent of deficits present). The frailty index was further divided into quintiles for comparison. A proportional hazards model was estimated for the MMD outcome controlling for other baseline information. A data driven approach was also used to determine relevant cut-offs in the frailty index where the trial intervention appeared to be modified. Among 1635 trial participants, the mean frailty index was 30.4 ± 6.6 and normally distributed. Over 2.5 years of average follow-up, 14.6%, 16.5%, 18.6%, 22.6%, and 27.6% of participants experienced MMD in quintiles 1–5, respectively. Each 1-unit increase in the frailty index increased the hazard of MMD by 4% (2–5%), and there was a nearly 2-fold increase in MMD between the highest and lowest frailty quintiles. Using log-rank criteria, a cut-point at the median was identified. Further, iterations tested for a frailty cut-off and indicated a subgroup beyond the 85th percentile wherein the physical activity intervention appeared to be no longer be effective. This internally derived deficit accumulation frailty index was uniquely able to identify individuals at higher risk of MMD and pMMD and showed that along the spectrum of frailty, the physical activity intervention remained effective for the majority of participants.

## 1. Introduction

Frailty is a geriatric syndrome represented by susceptibility to precipitating health events and reduced functional reserve [1,2,3]. Measuring frailty in older adults is challenging as both a baseline measure as well as an outcome in clinical research [1]. Efforts to measure or operationalize frailty in clinical research and practice can be summarized within two general approaches: phenotype assessment and deficit accumulation [1,2,4,5,6,7]. Phenotypic frailty assessments are typically criterion-based usually focusing on several components of physical, cognitive, psychological, or social functioning [2,6,8]. Deficit accumulation approaches assess a larger number of less specific measures such as the presence of comorbidities, physical or cognitive assessments, and lab tests, and are often summarized as a score or “index” proportional to the prevalence of possible deficits [6,7,8,9,10].

Efforts to develop measures of frailty have evaluated phenotypic or deficit accumulation frailty measures against a myriad of health outcomes. The widely used Fried frailty phenotype [2], consisting of unintentional weight loss, self-reported exhaustion, weakness, slow walking speed, and low physical activity, was shown to be predictive of falls, worsening functional status, hospitalization, and death. The Rockwood and Mitnitski Frailty Index approach [4], consisting of an index constructed from 70 possible deficits (e.g., presence of a comorbid disease constitutes a deficit), was shown to be associated with death and admission to a long-term care facility. Many other iterations of frailty measures have been described elsewhere [1]. Uniquely observed in most of the studies is that the measurement of either phenotypic or deficit accumulation frailty overlapped with but was not perfectly correlated with individual measures of functional status or multi-morbidity, and showed consistent ability of improving the prediction of clinical outcomes [1].

One goal of measuring frailty is to implement strategies to mitigate the effect of frailty on older adults. Studies into the prevention or reduction of frailty have included, among many others, physical activity (PA) interventions, such as that offered in the Lifestyle Interventions and Independence for Elders (LIFE) Study [11,12]. The primary outcomes in the LIFE Study were major mobility disability (MMD) and persistent MMD (MMD occurring for ≥12 months), operationalized as the inability to walk 400 m in 15 min. Frailty was not directly measured in the LIFE Study but several assessments of physical and cognitive functioning were performed at baseline and during follow-up. An important observation was that the baseline Short Physical Performance Battery (SPPB) scores modified the effect of the interventions in that those with lower SPPB scores (≤7 vs. 8–9) experienced a larger benefit of the physical activity intervention, suggesting that underlying functional status may modify the intervention effects [11]. In a secondary analysis of the LIFE Study, an existing frailty phenotype (the Study of Osteoporotic Fractures (SOF)) was applied to the LIFE Study cohort at baseline, and it was found that the intervention was not modified by this measure of baseline frailty nor was the SOF phenotype modified by the intervention [13]. Exploration of additional approaches to operationalize frailty in the context of the LIFE Study and PA interventions can be informative for future research and practice.

The primary objectives of this research were to develop a deficit accumulation frailty index in the LIFE Study cohort and to evaluate the index’s association with MMD and persistent MMD as robust, objective measures of physical functioning in older adults. A secondary objective, generated from prior observations that lower functional scores (e.g., SPPB ≤ 7) were associated with larger improvements in the LIFE Study, aimed to identify cut-points in the developed frailty index that may indicate groups who may benefit more from PA interventions.

## 2. Methods

### 2.1. LIFE Study Overview

The LIFE Study was a multi-center, single-blind, parallel randomized trial conducted across eight centers in the United States between February 2010 and December 2013 [14]. The study protocol was approved by the institutional review boards of each participating institution. Written informed consent was obtained from all study participants. The LIFE Study was registered with www.clinicaltrials.gov prior to participant enrollment in the trial (NCT01072500). Details of the study design, rationale, and characteristics of the full study population are described elsewhere [14,15].

### 2.2. Intervention

The PA intervention involved walking, with a goal of 150 min per week, strength, flexibility, and balance training. The intervention included attending two center-based visits per week and home-based activity three to four times per week for the duration of the study (3-years). The HE control intervention included weekly educational workshops during the first 26 weeks, and monthly sessions thereafter. Workshops included topics relevant to older adults, such as how to effectively negotiate the health care system, how to travel safely, preventive services and screenings recommended at different ages, where to go for reliable health information, and nutrition. The workshops did not include any topics related to exercise or physical activity.

### 2.3. Follow-Up Visits and Outcome Assessment

Participants were assessed every 6 months at clinic visits. Home, telephone, and proxy assessments were attempted if participants could not return to the clinic. The assessment staff were blinded to the intervention assignment and remained separate from the intervention team. Participants were asked not to disclose their assigned intervention arm or talk about their interventions during the assessments.

Details of MMD ascertainment were reported previously [12]. Briefly, participants were asked to walk 400 m at their usual pace, and MMD was defined as the inability to complete the walk within 15 min without sitting and without the help of another person or walker. When MMD could not be objectively measured because of the inability of the participant to come to the clinic and absence of a suitable walking course at the participant’s home, institution, or hospital, an alternative adjudication of the outcome was based on objective inability to walk 4 m in less than 10 s, or self-, proxy-, or medical-record-reported inability to walk across a room. If participants met these alternative criteria, they were considered to be unable to complete the 400-m walk within 15 min. Two consecutive MMD assessments or MMD followed by death defined persistent MMD (pMMD).

### 2.4. Deficit Accumulation Frailty Index Development

Baseline assessments in the LIFE Study included batteries of clinical history, laboratory measures, physical functioning assessments, and cognitive tests as well as quality-of-life and other surveys. A full list of the included variables is provided in the Supplementary Materials (Table S1), and performance of these assessments is described elsewhere [14].

To generate the frailty index, we followed the approach and recommendations of Searle et al. [16] and accumulated all relevant LIFE Study baseline assessments. For binary variables, deficits were assessed as being present or not and assigned a value of 0 or 1, respectively. For score-based assessments such as SPPB, grip strength, and surveys, participants were categorized into quintiles for each assessment. Weights were assigned to the deficit based on quintile assignment, with the best functioning quintile receiving a deficit of 0 and each subsequent quintile receiving a score of 0.25, 0.5, 0.75, or 1, respectively. A total of 75 possible deficits were ultimately identified, and the frailty index was operationalized as the total number of deficits present divided by the total number possible multiplied by 100, with the final value ranging from 0 (no deficits) to 100 (all deficits).

### 2.5. Statistical Analysis

We aimed to internally verify the performance of the frailty index based on its face validity and correlation with other baseline measures as well as with the primary outcomes in the LIFE Study. First, the cohort was divided into quintiles based on the frailty index, assigned so that lower quintiles had lower frailty indices, and described by baseline characteristics and assessments using group means and standard deviations, or proportions where appropriate. We further tested correlations of the frailty index with gait speed, grip strength, and the Short Physical Performance Battery assessments using Pearson correlation coefficients.

We further evaluated the primary outcomes of the LIFE Study, namely MMD and pMMD, and their association with the frailty index. A proportional hazards model was estimated controlling for age, gender, race, and baseline SPPB score. The frailty index was operationalized as both a continuous and a categorical measure. To identify relevant cut-points for the frailty index, we further implemented data-driven approaches and an “outcome-oriented” approach, which maximizes the log rank statistic using the method of Contal and O’Quigley [17] and the %FINDCUT macro [18]. We further manually iterated an interaction term between the intervention (PA vs. HE) and various quantiles (median, 75%, 85%, 90%, and 95%) of the frailty index with the aim to identify at what level, if any, of increasing frailty the PA intervention appeared to no longer be effective. Regression models were estimated for MMD and pMMD separately.

All analyses were performed in SAS v9.4 with a significance level of *p* < 0.05.

## 3. Results

**Figure 1:**
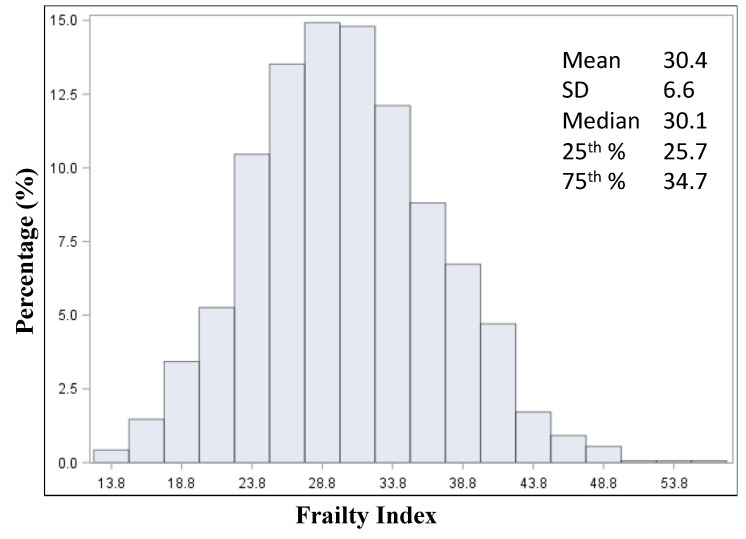
Distribution and summary statistics of the frailty index.

Among the 1635 participants, the mean age was 79 years, and 67.2% were female. The mean frailty index was normally distributed with a mean of 30.4 ± 6.6, a median of 30.1, an interquartile range of 25.7 to 34.7 (Figure 1), a minimum value of 14.1, and a maximum of 56.9. Baseline characteristics stratified by quintiles are summarized in Table 1.

A higher frailty index was associated with worse baseline assessments and presence of comorbid conditions, which was expected, as these assessments were part of the frailty index. Notable differences were a 3-fold difference in reported “worsening health of last 6 months” for those with lowest frailty (Q1: 4.6%) versus those with highest frailty (Q5: 18.0%). Similarly, there was a 6-fold difference in overall self-reported health status, with 6.1% of those in Q1 reporting “bad health” compared with 37.9% of those in Q5. Among other notable disparities in baseline conditions, 84.7% of those in the highest frailty group reported fatigue compared with 18.0% in the lowest quintile. Age was not significantly associated with quintiles of frailty but was associated with a higher proportion of female gender, with 54.4% of those in Q1 but 74.9% of those in Q5 being female.

**Figure 2:**
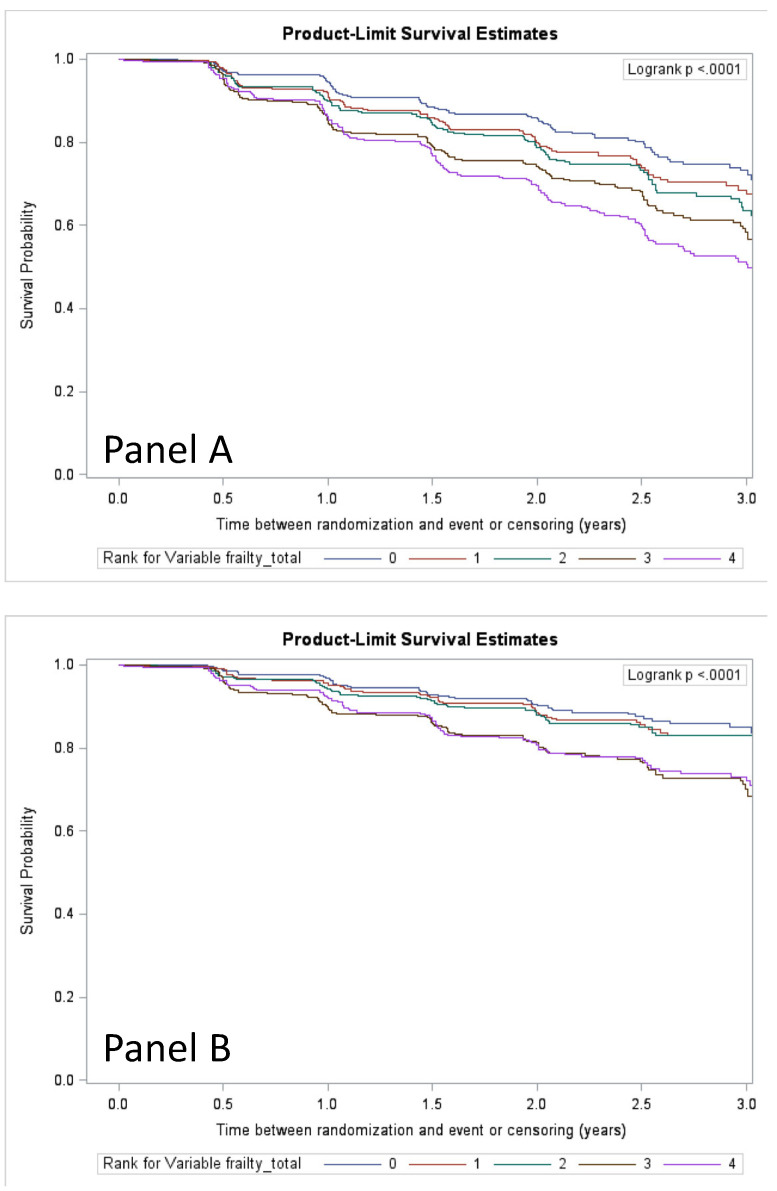
Kaplan-Meier plots of major mobility disability (MMD) (Panel **A**) and persistent MMD (pMMD) (Panel **B**) for quintile-stratified frailty index groups over 3 years of follow-up. Quintile 0 is equivalent to the group with the least frailty measures and Quintile 4 is the group with the most.

**Figure 3:**
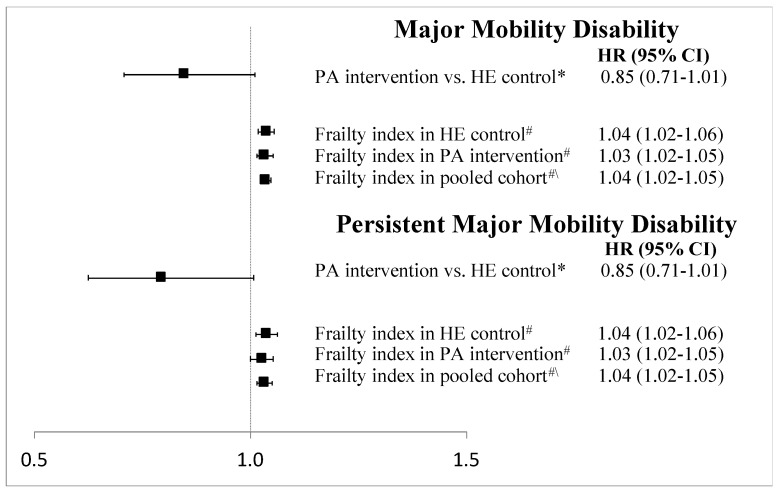
Cox proportional hazard regression results of the interaction between the LIFE Study intervention and a continuous frailty index for both major mobility disability and persistent major mobility disability. * Results show the overall effect of the physical activity (PA) intervention compared with the health education (HE) control arm at the mean frailty index. ^#^ Per 1-unit change in frailty index. ^#\^ Results for the overall pooled cohort.

**Figure 4:**
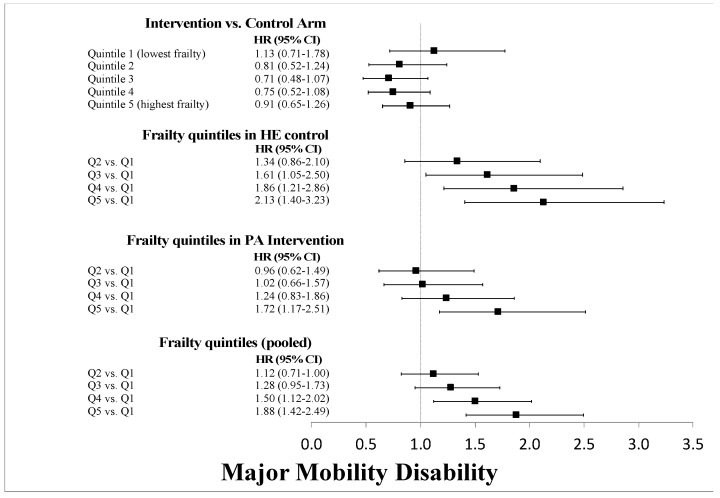
Cox proportional hazard regression results for the interaction between the LIFE Study intervention and quintile stratification of the frailty index for major mobility disability. The topmost results compare the physical activity (PA) intervention versus the health education (HE) control arm stratified by frailty quintiles. The stratified and pooled associations of the quintiles are also shown.

**Figure 5:**
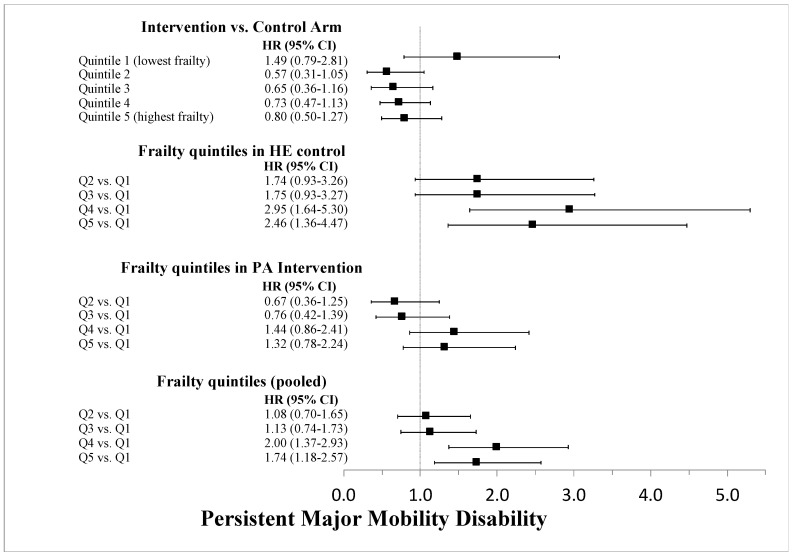
Cox proportional hazard regression results for the interaction between the LIFE Study intervention and quintile stratification of the frailty index for persistent major mobility disability. The topmost results compare the physical activity (PA) intervention versus the health education (HE) control arm stratified by frailty quintiles. The stratified and pooled associations of the quintiles are also shown.

Over 2.5 years of average follow-up, 14.6%, 16.5%, 18.6%, 22.6%, and 27.6% of participants experienced MMD in Q1 through Q5, respectively. Kaplan-Meier survival plots of MMD and pMMD outcomes stratified by frailty quintiles are shown in Figure 2. In regression models (Figure 3), each 1-unit increase in the frailty index increased the hazard of MMD by 4% (2–5%), with similar results for pMMD. Stratified by quintiles, there was a nearly 2-fold increase in both MMD and pMMD between the highest and lowest quintiles of frailty (Figure 4 and Figure 5). Overall, the HE control arm showed stronger associations with MMD and pMMD with increasing frailty.

**Figure 6:**
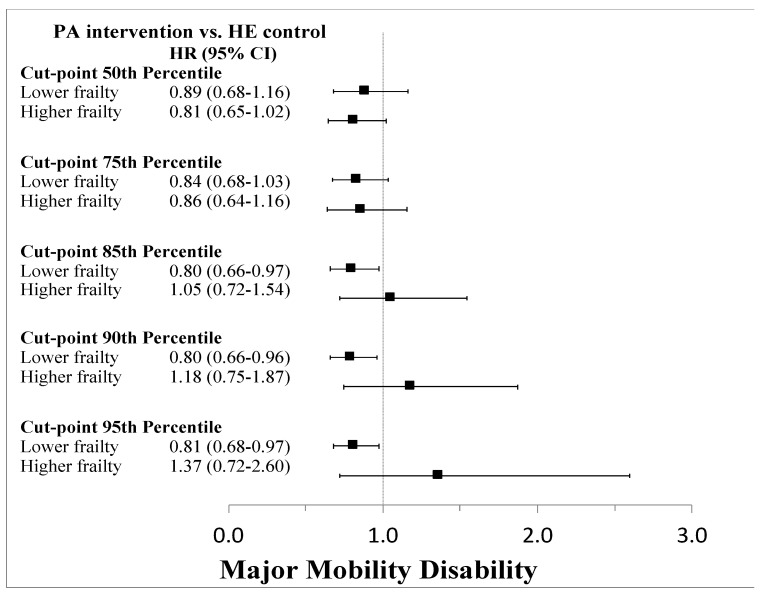
Cox proportional hazards regression results testing frailty index cut-points as strata to identify subgroups in which the intervention was no longer effective in preventing major mobility disability.

**Figure 7:**
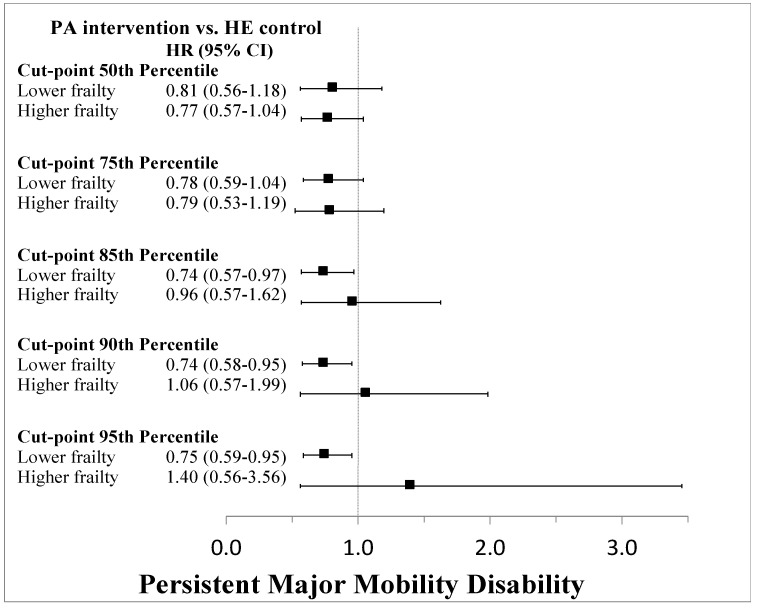
Cox proportional hazards regression results testing frailty index cut-points as strata to identify subgroups in which the intervention was no longer effective in preventing persistent major mobility disability.

Data driven approaches using the Cogan and O’Quigley method identified a relevant cut-point at roughly the median value (around 30). Thus, a binary frailty index status divided the cohort into those below or above this median but found no difference in the effectiveness of the PA intervention compared to the HE control. Additional cut-points were evaluated at the 75th, 85th, 90th, and 95th percentiles and found that there were differences between the PA and HE groups apparent at the 85th percentile and above for both MMD and pMMD (Figure 6 and Figure 7). Output from the data-driven cut-point selection processes automated by the SAS macro %FINDCUT are presented in Figure S1 (Supplementary Materials). Kaplan-Meier survival curves for each cut-point stratified by intervention group assignment are shown in Figure S2 (Supplementary Materials). Pearson correlation coefficients for the association of the frailty index with SPPB, grip strength, and gait speed revealed overall poor correlations (Table 2 and Figure S3 (Supplementary Materials)).

## 4. Discussion

Classifying frailty using a deficit accumulation approach is attractive for risk stratification and prediction of future health events. In this analysis, the frailty index was a strong independent predictor overall for risk of MMD but did not have a clear interaction with the intervention. However, the 85th percentile was determined as a minimum cut-off that distinguished a subgroup that did not respond to the PA intervention, which is an important finding for several reasons. First, it shows that for a majority of individuals, the intervention was effective. Second, it facilitates a comparison with the original trial results that showed that those with SPPB ≤ 7 were more responsive to the intervention [11,12]. Thus, it demonstrates further utility of the frailty index as a tool to differentiate beyond commonly used geriatric assessments like SPPB. In fact, the frailty index derived in this study had poor correlation with baseline SPPB scores, grip strength, and gait speed, and provides evidence that further risk stratification is possible using such measures.

The use of frailty index developed via deficit accumulation approaches has been used and validated for the prediction of future mortality among many other outcomes and compared with the frailty phenotype as well. Using data from the Canadian Study of Health and Aging and a cohort of patients with some cognitive impairment, Rockwood et al. combined 70 possible deficits to develop a frailty index and compared it with phenotypical measurements of unintended weight loss, exhaustion, low physical activity levels, weakness, and slowness [6]. While they reported results stratified by presence of phenotypic frailty, their findings appear similar to ours, with a median frailty index near 30, a strong correlation and agreement with the frailty phenotype, and ability to stratify the cohort for risk of mortality and institutionalization.

Our study is unique in that we evaluated the impact of the frailty index on MMD as well as its interaction with a physical activity intervention in older adults. While our results suggest a strong association between frailty and MMD, there was little interaction between the intervention and the frailty index until very high levels of frailty (85th percentile and above). We evaluated MMD, as it captures physical mobility in older adults, which is an important component of living independently. The promotion of healthy aging and mobility through physical activity and other interventions has the potential to reduce healthcare events, utilization, and costs among the growing population of older adults at risk of physical functioning limitations [19]. Developing analytical techniques to identify those who may respond better to such interventions is one use of frailty indices, and further work is needed to validate their utility in clinical research and practice.

The use of the derived frailty index is limited, as many of the variables were only collected at baseline or intermittently throughout the trial. Thus, the frailty index could not be measured as a dynamic variable or outcome throughout the trial. There is little current information but further interest in how baseline frailty is associated with future frailty and how changes in frailty are associated with outcomes such as death. In one study including up to 54 months of follow-up, Fallah et al. showed that baseline frailty was predictive of future changes in frailty [20]. Further, when frailty was incorporated into mortality outcome models, the effect of mobility was not significant for mortality. Thus, understanding the dynamic relationship between frailty and mobility as it relates to mortality is a focus of future research with the LIFE Study cohort. The observation that baseline frailty was less strongly associated with pMMD in the current study also implies differences in what the frailty index captures. For instance, a strong association with MMD but not pMMD may suggest frailty indices predict incident health events but are less predictive of an individual’s ability to recover from these incident health events. These nuances deserve future study to understand what is being captured by a frailty index and the interplay with mobility, intervening health events, recovery, and mortality.

Frailty indices are internally derived and dependent on variables unique to each study [16]. Thus, the frailty index developed and validated here is likely not applicable to other cohorts without similar measures. Frailty indices are also composites of a large number of variables available in a dataset. They may have overlap in the domain being measured and could have a significant correlation. Paring down a frailty index to a minimum number of variables is of interest in future research to derive a minimum index with similar predictive validity. Lastly, the LIFE Study cohort is representative of an older adult population of ≥70 years of age with existing mobility concerns, and the observed results regarding the intervention and impact of frailty may not be generalizable to the general population of older adults. Regardless, this work shows how frailty indices can be applied to understand the dynamic relationships between interventions, other assessments of physical and cognitive functioning, and important outcomes such as MMD in older adults.

## 5. Conclusions

We developed a deficit accumulation frailty index and evaluated its association with MMD and interaction with a physical activity intervention in a study with older adults. Our results show strong discrimination between those at risk of physical mobility limitations and demonstrate that the frailty index uniquely captures variability in MMD risk compared to other physical assessments included in the study. Operationalizing frailty via a deficit accumulation approach appears to be a valid measure of frailty in older adults and may have a role in both clinical research and practice.

## Figures and Tables

**Table 1:** Distributions of baseline demographics, clinical characteristics, and physical assessments stratified by quintiles of the frailty index.

Frailty Index Quintiles	Q1 (Least Frail)		Q2		Q3		Q4		Q5 (Most Frail)	
Variable Name	N/ mean	%/SD	N/ mean	%/SD	N/ mean	%/SD	N/ mean	%/SD	N/ mean	%/SD
PA intervention assignment	179	54.7	158	48.6	163	49.5	165	50.5	168	51.4
Age (years)	79.4	5.3	79.1	5.1	78.5	5.3	78.9	5.3	78.5	5.1
Education ≥ High school	233	71.3	223	68.6	218	66.3	225	68.8	207	63.3
Female gender	178	54.4	215	66.2	229	69.6	239	73.1	245	74.9
Race										
Black	67	20.6	57	17.7	63	19.6	41	12.6	55	17.2
Other	22	6.8	22	6.8	21	6.5	15	4.6	27	8.4
White	236	72.6	243	75.5	238	73.9	269	82.8	238	74.4
Self-reported health status										
Bad or poor health	20	6.1	32	9.9	38	11.6	61	18.7	124	37.9
Health worsened last 6 months	15	4.6	21	6.5	23	7.0	45	13.8	59	18.0
Past medical history										
Overweight	99	30.3	150	46.2	168	51.1	151	46.2	184	56.3
High blood pressure	190	58.1	215	66.2	236	71.7	250	76.5	264	80.7
Heart attack	12	3.7	20	6.2	24	7.3	41	12.5	65	19.9
Heart failure	9	2.8	9	2.8	13	4.0	28	8.6	41	12.5
Stroke	14	4.3	17	5.2	30	9.1	25	7.7	34	10.4
Cancer	62	19.0	62	19.1	80	24.3	82	25.1	106	32.4
Diabetes	50	15.3	79	24.3	85	25.8	103	31.5	135	41.3
Broke hip	20	6.1	16	4.9	16	4.9	7	2.1	22	6.7
Arthritis	28	8.6	53	16.3	67	20.4	67	20.5	107	32.7
Back injury	19	5.8	28	8.6	29	8.8	45	13.8	75	22.9
Fainting	6	1.8	13	4.0	18	5.5	23	7.0	45	13.8
Anxiety	32	9.8	47	14.5	70	21.3	109	33.3	174	53.2
Fatigue	59	18.0	134	41.2	162	49.2	223	68.2	277	84.7
Decreased appetite	19	5.8	28	8.6	34	10.3	62	19.0	89	27.2
Insomnia	38	11.6	72	22.2	88	26.8	136	41.6	168	51.4
Dizziness	21	6.4	49	15.1	53	16.1	89	27.2	135	41.3
Muscle stiffness	96	29.4	160	49.2	131	60.2	95	71.0	60	81.7
Foot pain	42	12.8	66	20.3	85	25.8	113	34.6	149	45.6
Previous hospitalization	8	2.5	22	6.8	17	5.2	31	9.5	56	17.1
Angina	15	4.6	27	8.3	34	10.3	37	11.3	75	22.9
Transient ischemic attach	19	5.8	34	10.5	53	16.1	55	16.8	83	25.4
Physical assessments										
CHAMPS	21.2	35.5	18.8	35.0	18.2	33.8	16.9	32.8	10.4	26.4
ADL limitations	1.1	0.2	1.2	0.3	1.3	0.4	1.4	0.4	1.6	0.4
IADL limitations	1.0	0.1	1.1	0.2	1.1	0.3	1.1	0.3	1.3	0.4
Gait speed (m/s)	0.9	0.2	0.8	0.2	0.8	0.2	0.8	0.2	0.8	0.1
Number of medications	3.4	2.5	4.3	2.6	5.0	2.9	5.4	3.2	6.7	3.5
Grip strength (kg)	26.6	10.7	24.4	10.3	23.4	9.4	23.4	9.8	22.1	8.6
Total steps at moderate pace	1381.9	1467.4	1058.9	1143.7	948.4	1008.3	880.2	858.5	617.0	571.7
SPPB score	7.6	1.7	7.5	1.6	7.4	1.5	7.2	1.6	7.2	1.5

Abbreviations: ADLs = activities of daily living; IADLs = instrumental activities of daily living; SPPB = short physical performance battery; kg = kilograms; m/s = meters per second; CHAMPS = Community Health Activities Model Program for Seniors score; PA = physical Activity intervention.

**Table 2:** Correlation between increasing baseline frailty index and other baseline physical functioning assessments.

	SPPB Score	Gait Speed (m/s)	Grip Strength (kg)
Pearson correlation coefficient (r)	−0.104	−0.218	−0.145

SPPB = Short Physical Performance Battery score; m/s = meters per second; kg = kilograms.

## Supplementary Materials

The following are available online at https://www.mdpi.com/2077-0383/9/11/3757/s1, Table S1: LIFE Study variable names included in the frailty index, Figure S1: Output from the %FINDCUT macro program applying the Cogan and O’Quigley method to identify a cut-point in the frailty index based on data-driven measures, Figure S2: Kaplan-Meier plots of survival probabilities for MMD stratified by cut-point iterations at the 50th, 75th, 85th, 90th, and 95th percentiles and by intervention or control group assignment, Figure S3: Correlation plots of the frailty index (*y*-axis) with various physical functioning measures (*x*-axis) including Short Physical Performance Batter score, grip strength, and gait speed.
