# Supplementary material for: Association between a Deficit Accumulation Frailty Index and Mobility Outcomes in Older Adults: Secondary Analysis of the Lifestyle Interventions and Independence for Elders (LIFE) Study
Source: J Clin Med. 2020 Nov 22;9(11):3757. doi: 10.3390/jcm9113757 (PMC7700674; doi:10.3390/jcm9113757)
Supplement: Supplementary file 1 [file jcm-09-03757-s001.pdf]

Table S1: LIFE Study variable names included in the frailty index

**Binary Measures (yes/no) assigned values of 0 or 1 based on presence**	
Underweight (BMI ≤18.5)	Fatigue
Obese (BMI ≥30)	Decreased appetite
Poor health (self-rated)	Insomnia
Health worsened last 6 months (self-rated)	Dizziness
High blood pressure	Stiff muscles
Heart attach	Torn muscles
Heart failure	Foot pain
Pacemaker	Hospitalized in last 6 months
Stroke	Peripheral artery disease
Cancer	Asthma
Diabetes	Pneumonia
Broken hip	Bronchitis
Broken bone	Low vision
Arthritis	CABG history
Liver disease	
Psychiatric conditions	
Other conditions	
Back injury	
Paralysis	
Fainting	
Abnormal heart rhythm	
Foot ulcer	
Wounds	
Anxiety	

Abbreviations: BMI=body mass index; CABG=coronary artery bypass grafting

**Continuous or discrete numerical variables assigned weights (0, 0.25, 0.5, 0.75, 1) based on quintiles**	
Baseline SPPB	Total Rey-O
CHAMPS score	MATSF Score
Basic Well-being	Minutes of moderate activity
Pepper Assessment Tool for Disability	Steps per day
Mobility Well-being	
I/ADLs	
Baseline gait speed	
Self-efficacy for walking 400m	
CPX	
MOB	
PAC	
SAC	
TotalQWB	
Number of medications	
Grip strength	
CESD Score	
HRQL Stress	
3MSE	
Cognitive battery test scores (9)	
Epworth Sleep	
Insomnia score	
Pitt Sleep Score	
Berlin score	
Sum FAQ Score	

Abbreviations:
SPPB=short physical performance battery; CHAMPS=Community Health Activities Model Program for Seniors; I/ADLs=instrumental/activities of daily living ; CPX=Symptoms score from the QWB survey; MOB=Mobility score from the QWB survey; PAC= Physical activity score from the QWB survey; SAC=Social & self-care activity score; QWB=Quality and Wellbeing Survey total score ; CESD=Center for Epidemiologic Studies Depression scale; HRQL=health-related quality of life ; 3MSE=Modified Mini-Mental State Examination; FAQ=Functional Activities Questionnaire ; Rey-O=Rey-Osterrieth Complex Figure assessment; MATSF= Mobility Assessment Tool-short form;

Figure S1: Output from the %FINDCUT macro program applying Cogan and O'Quigley's method to identify a cut-point in the frailty index based on data driven measures

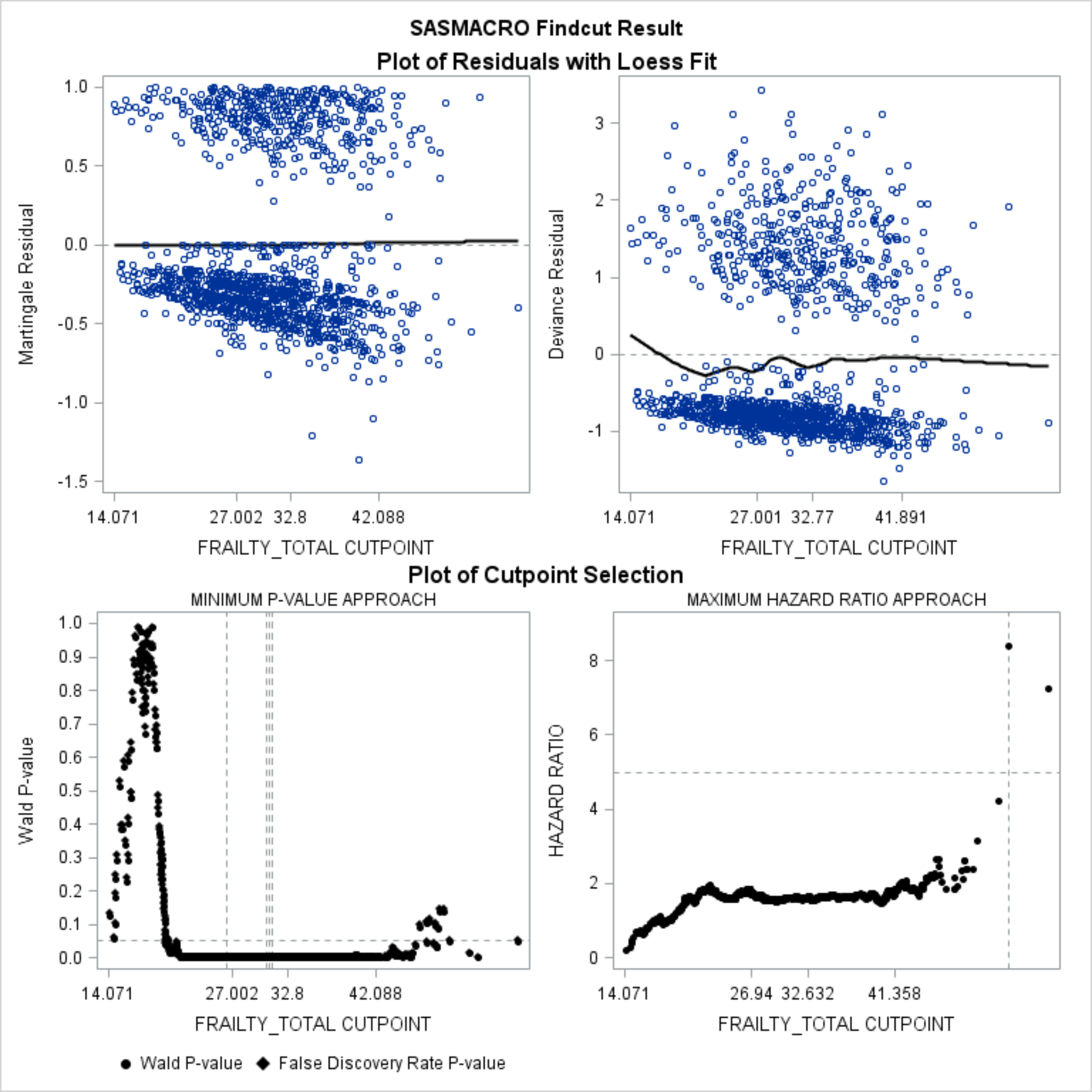

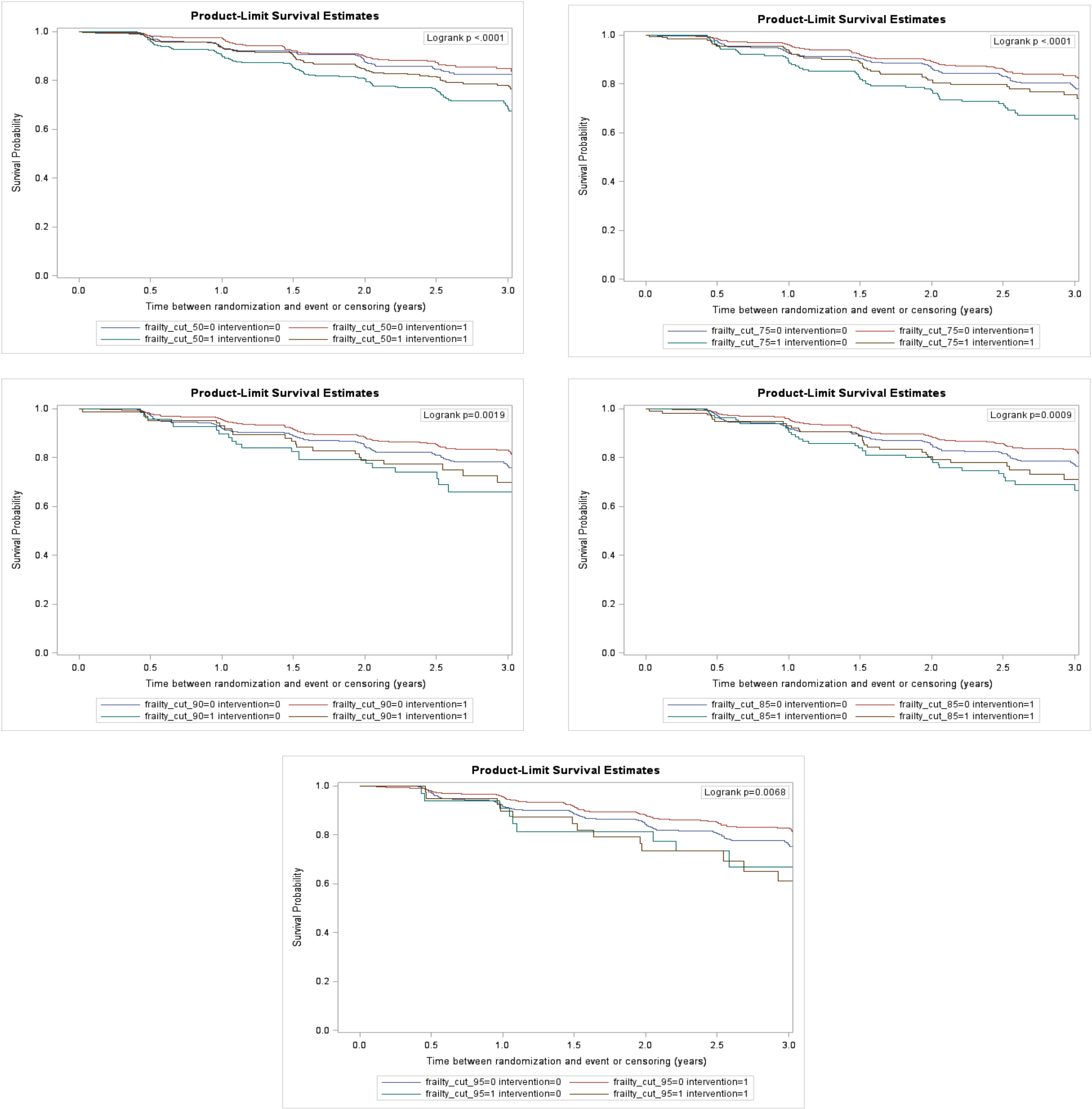

Figure S2: Kaplan-Meier plots of survival probabilities for MMD stratified by cut-point iterations at the 50th, 75th, 85th, 90th, and 95th percentiles and by intervention or control group assignment.

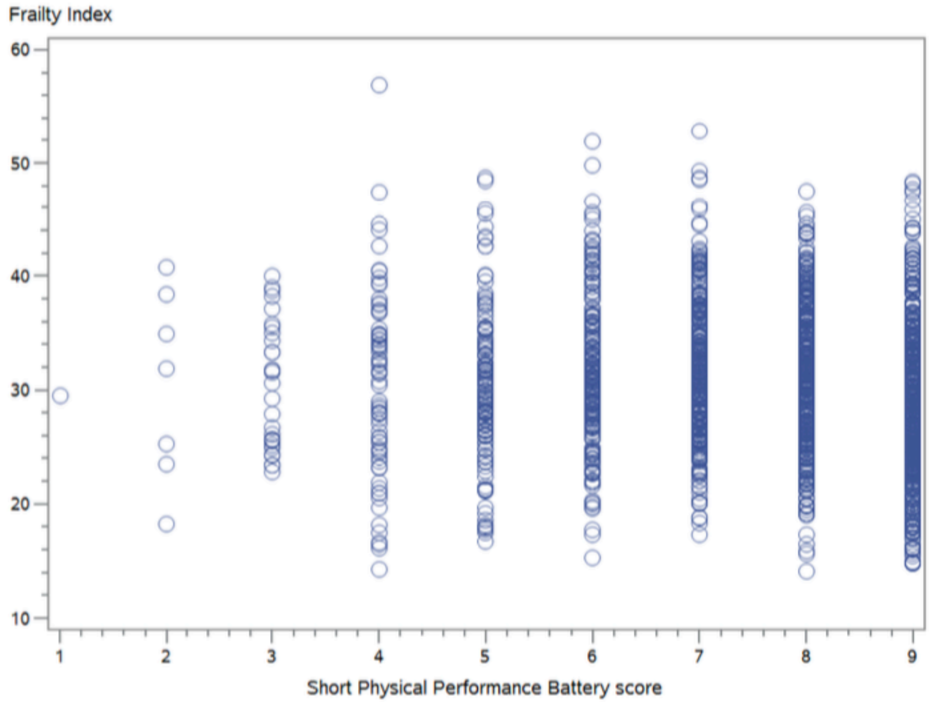

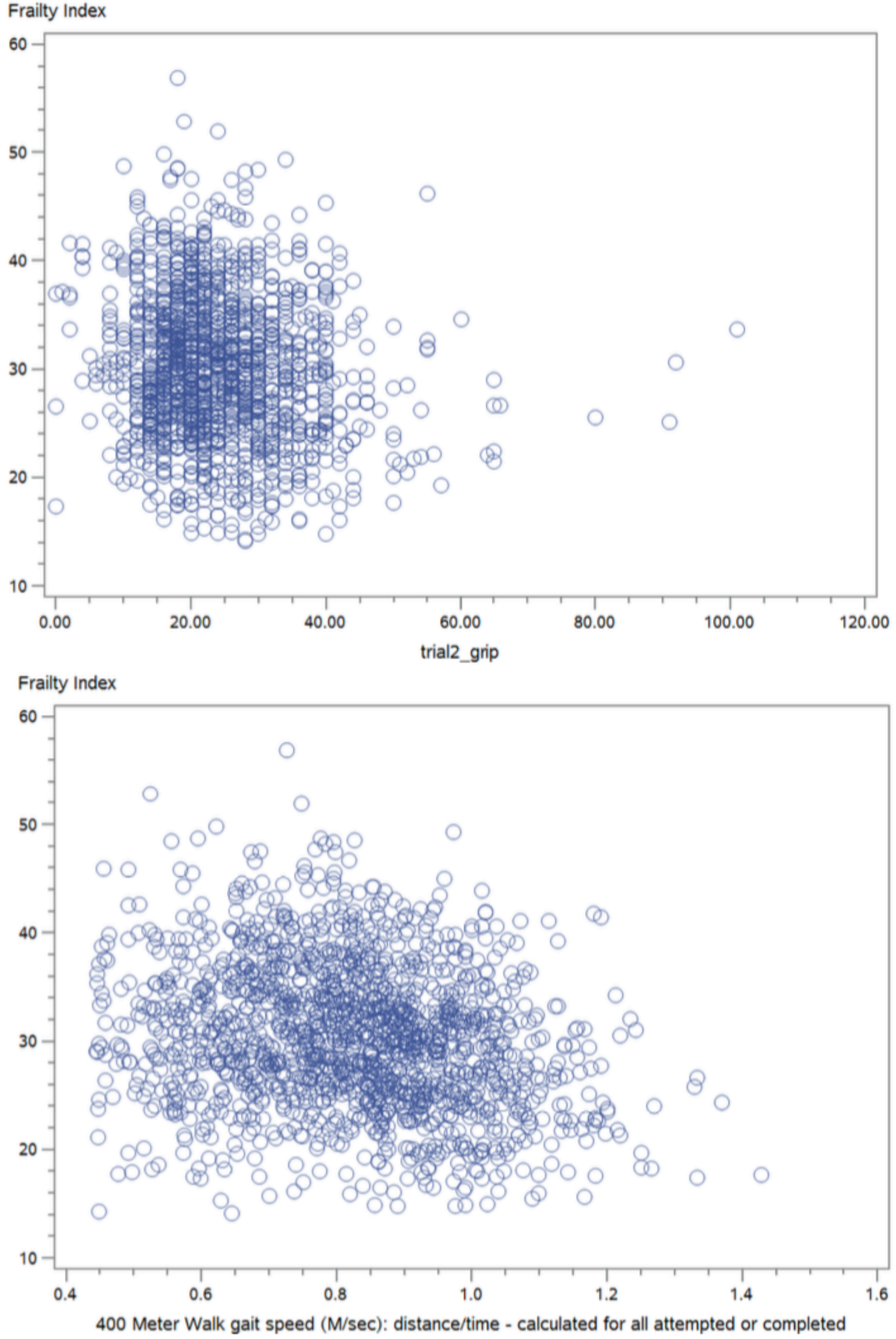

Figure S3: Correlation plots of the frailty index (y-axis) with various physical functioning measures (x-axis) including Short Physical Performance Batter score, grip strength, and gait speed.
